# Systematic analysis of the pectin methylesterase gene family in *Nicotiana tabacum* and reveal their multiple roles in plant development and abiotic stresses

**DOI:** 10.3389/fpls.2022.998841

**Published:** 2022-09-28

**Authors:** Jinhao Sun, Zhen Tian, Xiaoxu Li, Shaopeng Li, Zhiyuan Li, Jinling Wang, Zongyu Hu, Haiqing Chen, Cun Guo, Minmin Xie, Ruyan Xu

**Affiliations:** ^1^ Technology Center, China Tobacco Jiangsu Industrial Co., Ltd., Nanjing, China; ^2^ Key Laboratory for Tobacco Gene Resources, Tobacco Research Institute, Chinese Academy of Agricultural Sciences, Qingdao, China; ^3^ Kunming Branch of Yunnan Provincial Tobacco Company, Kunming, China

**Keywords:** tobacco, PME genes, genome-wide analysis, abiotic stresses, root development

## Abstract

The pectin methylesterases (PMEs) play multiple roles in regulating plant development and responses to various stresses. In our study, a total of 121 *PME* genes were identified in the tobacco genome, which were clustered into two groups based on phylogenetic analysis together with *Arabidopsis* members. The investigations of gene structure and conserved motif indicated that exon/intron and motif organizations were relatively conserved in each group. Additionally, several stress-related elements were identified in the promoter region of these genes. The survey of duplication events revealed that segmental duplications were critical to the expansion of the *PME* gene family in tobacco. The expression profiles analysis revealed that these genes were expressed in various tissues and could be induced by diverse abiotic stresses. Notably, *NtPME029* and *NtPME043*, were identified as homologues with *AtPME3* and *AtPME31*, respectively. Furthermore, *NtPME029* was highly expressed in roots and the over-expression of the *NtPME029* gene could promote the development of roots. While *NtPME043* could be induced by salt and ABA treatments, and the over-expression of the *NtPME043* gene could significantly enhance the salt-stress tolerance in tobacco. Overall, these findings may shed light on the biological and functional characterization of *NtPME* genes in tobacco.

## Introduction

The plant cell walls mainly contain structural proteins and three types of polysaccharides, including cellulose, hemicellulose, and pectins (Zhao et al., 2019). Pectins as highly heterogeneous polymers exist between the intercellular layer and the primary wall and it accounts for 30-40% of the cell wall of dicots and non-graminaceous monocots. The pectin modification leads to crucial changes in plant cell walls’ structure and function, affecting plant development, including fruit ripening and softening, organ abscission and senescence, pollen dehiscence, pollen tube growth, and stress resistance (Al-Qsous et al., 2004; Bosch et al., 2005; An et al., 2008; Zhang et al., 2010; Verma et al., 2014; Wallace and Williams, 2017). Pectins are synthesized with a highly methylesterified form and then are catalyzed to decrease methyl esterification by a large enzyme family of pectin methylesterases (PMEs) (EC. 3.1.1.11) that reside in the cell wall (Yapo et al., 2007; Wolf et al., 2009).

In plants, PMEs can be divided into two groups (Type I and Type II) according to whether it contains the PMEI (pectin methylesterase inhibitor domain) domain. Type I (PME type) contain the PME domain and the pro-region of its N-terminal, which is very short or missing. However, type II (ProPME type) contains simultaneously PME and PMEI domain and a long N-end pro-region (Udall et al., 2006). The pro-region was reported to fold the protein correctly and inhibit the PME activity (Micheli, 2001; Pelloux et al., 2007). Previous research has reported that there are five conserved structure regions, including Region I (GxYxE), Region II (QAVAxR), Region III (QDTL), Region IV (DFIFG), and Region V (YLGRxWx) in the secondary structure of PME proteins (Markovic and Janecek, 2004). The Region I is conserved in all PME proteins, while the remaining regions have diverse conservation in different plants.

A total of 66 PMEs have been identified in the *Arabidopsis*, 22 members falling into type I and 44 into type II, and the contributions of some PMEs to various developmental processes have been characterized (Tian et al., 2006; Levesque-Tremblay et al., 2015). For example, *AtPME6* and *AtPME58* express highly during mucilage secretion, which is involved in embryo development and mucilage extrusion, respectively (Levesque-Tremblay et al., 2015; Turbant et al., 2016). In addition, the knockout mutants of *AtPME1* specifically exhibit curved, irregular morphology and are significantly stunted in the pollen tube compared that with wild type (Tian et al., 2006). *AtPME48* specifically expressed in the male gametophyte and functioned to influence pollen grain germination (Leroux et al., 2015). Furthermore, *AtPME5*-overexpressing transgenic plants were reported to increase the cell walls elasticity in shoot apical meristem (Peaucelle et al., 2011). *AtPME35* influences the mechanical strength of the stem by regulating the PME demethylesterifification (Hongo et al., 2012). *AtPME17* is significantly expressed in vegetative tissues and could be processed by SBT3.5 (subtilisin-type serine protease 3.5) to modify PME activity which is involved in root growth (Sénéchal et al., 2014). Moreover, *AtPME3*-overexpressing plants showed longer roots compared to wild-type, whereas the RNAi plants showed the opposite phenotype (Hewezi et al., 2008).

Besides, many PMEs facilitate responses to multiple biotic and abiotic stresses in *Arabidopsis*. Over-expressed *AtPME31* provides broad-spectrum insect resistance in transgenic tobacco plants (Dixit et al., 2013). Moreover, *AtPME31* could be significantly induced under salt stress and positively modulate the expression levels of salt stress-induced genes to enhance the salt stress tolerance of plants (Yan et al., 2018). *AtPME34* is proposed that responds to heat and salt stress through ABA (abscisic acid) pathway (Huang et al., 2017). It was verified that *AtPME41* contributes to freezing tolerance by modulating the mechanical properties of cell walls through the BR (brassinosteroid) signaling (Qu et al., 2011).

Tobacco is one of the most significant economic crops and widely cultivated all over the world, while various abiotic stresses can severely threaten tobacco quality and yield. The *PME* genes have been reported to be involved in response to abiotic stresses and affect development in a variety of species. Although the PME family proteins have been characterized in several plants (Duan et al., 2016; Zega and D'Ovidio, 2016; Zhang et al., 2019; Li et al., 2020), limited information is available about PME family proteins in tobacco. In this study, a comprehensive analysis was performed, including phylogenetic analyses, gene structure, motif organization, *cis*-elements, chromosomal distributions, duplication events, and expression profiles. The results of the current study imply that the tobacco NtPME family proteins play multiple roles in various biological processes.

## Materials and methods

### The identification and phylogenetic analysis of NtPME proteins

The full-length protein sequences for each AtPME in *Arabidopsis* were downloaded from TAIR (The *Arabidopsis* Information Resource) database (http://www.arabidopsis.org/), while the genomic data for tobacco (*N. tabacum*V1.0) were obtained from the SGN (Sol Genomics Network) database (https://solgenomics.net/). Then, the protein sequences of AtPME were used as queries to detect the potential NtPME proteins in tobacco by the BLASTp searches (E-value < 0.01) (Li et al., 2021). Subsequently, the potential sequences were examined using the SMART (Letunic et al., 2015) and PFAM (Finn et al., 2016) databases to determine the PME domains. The remaining NtPME numbers were renamed according to their physical locations on the chromosome or scaffold. The Mw (molecular weight) and theoretical pI (isoelectric points) of these finally identified NtPME numbers were predicted by ProtParam toolkits (Garg et al., 2016). The sequences of NtPME and AtPME proteins were aligned using Clustal X (Larkin et al., 2007), and the alignments were used to construct the neighbor-joining (NJ) tree by MEGA 6 with the default parameters method (Kumar et al., 2018). The subcellular localization of NtPME proteins was also predicted by Cell-PLoc 2.0 and WOLF POSRT II (https://www.genscript.com/psort.html) (Ren et al., 2019).

### Chromosomal localization and duplication event analysis of *NtPME* genes

The chromosomal location image of *NtPME* genes was visualized by MG2C, according to the data obtained from the SGN database (Chao et al., 2015). The segmental duplication events were identified and visualized by the MCScanX program and Circos, respectively (Cao et al., 2016). The syntenic analysis of the orthologous genes obtained from tobacco and other four plant species was investigated by Synteny Plotter (Xie et al., 2018). Then, the synonymous substitution (Ks) and non-synonymous substitution (Ka) rates were calculated by DnaSP 5.0 software (Librado and Rozas, 2009).

### Gene structure, conserved motifs, and *cis*-elements analyses of NtPME proteins

The gene structure of *NtPME* genes was analyzed and visualized using GSDS (http://gsds.cbi.pku.edu.cn) according to their genomic and coding sequence (Hu et al., 2015). The Multiple Em for Motif Elicitation (MEME) online tool was adopted to identify and visualize conserved motifs of the NtPME full-length protein sequences (Bailey et al., 2015). The promoter regions, 2000-bp sequences upstream of tobacco *NtPME* genes, were obtained by TBtools software (Chen et al., 2020), and then the *cis*-regulatory elements of these promoters were analyzed by the PlantCARE tool (Lescot et al., 2002).

### Expression analysis of NtPME genes in tobacco

The RNA-seq data for each tissue of *NtPME* genes in tobacco K326 were obtained from the GEO database (accession number: GSE95717) (Edwards et al., 2017). The absolute transcript abundance values of three tissues (root, shoot, and apex) of the *NtPME* genes were normalized and illustrated using R.

### Tobacco plant growth and stress treatments

The Cultivated tobacco K326 (*Nicotiana tabacum* L. Cv. K326) was used in the current study. The tobacco plants were cultivated in the growth conditions described in the previous report (Li et al., 2021). Different tissues, including the shoots, roots, leaves, and flowers were harvested to explore tissue-specific expression patterns and visualized in a heat map by TBtools (Wu et al., 2020). For abiotic stress (salt and ABA) treatments, the seedlings were treated with 50 µM Abscisic Acid (ABA) or 150 mM NaCl, and then harvested at 0, 1, 3, or 6 h after treatment (Sun et al., 2021). All the collected samples were frozen by the liquid nitrogen and then stored at -80 °C for RNA extraction.

### RNA extraction and RT-qPCR analysis

Total RNA was extracted and 1 µg RNA was synthesized to the first-strand complementary DNA (cDNA) by the method previously reported (Li et al., 2019a; Ren et al., 2019). The qRT-PCR was carried out on an ABI7500 Real-Time PCR System (Applied Biosystems, Foster City, CA, United States) with 2.5 µL template cDNA. The ribosomal protein gene *L25* (GenBank No. L18908) of tobacco was adopted as the internal control. All reactions were performed with three biological replications and the resulting data were evaluated by the 2 ^−ΔΔCT^ method (Livak et al., 2001). The specific relative primer sequences were designed by Primer Premier 5.0 and the details were shown in **Supplementary Table S1**.

### Subcellular localization

The coding regions of *NtPME029* and *NtPME043* were amplified by PCR with specific cloning primers and then generated into the *p*CHF3-GFP vector. The *NtPME029*-*GFP* and *NtPME043*-*GFP* fragments were driven by the *CaMV*-*35S* promoter, and the control was the GFP fragment driven by the *CaMV*-*35S* promoter. Subsequently, these constructs were injected into *Nicotiana benthamiana* leaves separately for transient expression as previously reported (Li et al., 2019b). After 3 days of growth in a light condition, the GFP fluorescence signals in these leaves were monitored by using the confocal microscope (TCS-SP8 Leica, Wetzlar, Germany) (Li et al., 2018).

### Overexpression analysis

The coding sequences of *NtPME029* and *NtPME043* were amplified and then generated into the expression vector (*p*CAMBIA1300). The *NtPME029* and *NtPME043* over-expression vectors were transformed into K326 tobacco plants by an *Agrobacterium*-mediated method and the empty vector as the control (Buschmann, 2016). The T0-generation seeds were screened in half-strength MS medium with 20 mg/L of hygromycin to obtain T1 seeds as the previous report (Li et al., 2021). The one-week-old T1 plants and wild-type tobacco plants were transferred to MS medium with or without 150 mM NaCl to grow for two weeks. Subsequently, the changes in root length were statistically analyzed.

### Statistical analysis

Student’s *t*-tests in GraphPad Prism 8 revealed significant differences from the control. *P* values less than 0.01 were considered significant. All data were analyzed in three replicates.

## Results

### Identification of *PME* genes in tobacco

To identify *PME* family genes in tobacco, we used the PME numbers of *Arabidopsis* as references to the BLASTP search. A total of 121 *PME* genes were identified in tobacco and named *NtPME001* to *NtPME121* based on their physical locations on chromosomes or scaffolds (**Supplementary Table S2**). Subsequently, the biochemical characteristics of *PME* genes were analyzed. As shown in **Supplementary Table S2**, the ORF (open reading frame) lengths ranged from 348 bp (*NtPME037*) to 3348 bp (*NtPME84*) and their protein weights (MWs) ranged from 12.63 (NtPME037) to 124.45 kDa (NtPME84). The isoelectric points (PIs) values of different NtPME proteins ranged from 4.30 (NtPME087) to 10.47 (NtPME034). The prediction of subcellular localization indicated that all NtPME proteins were located on the cell wall.

### Phylogenetic analysis of the NtPME members

**Figure 1:**
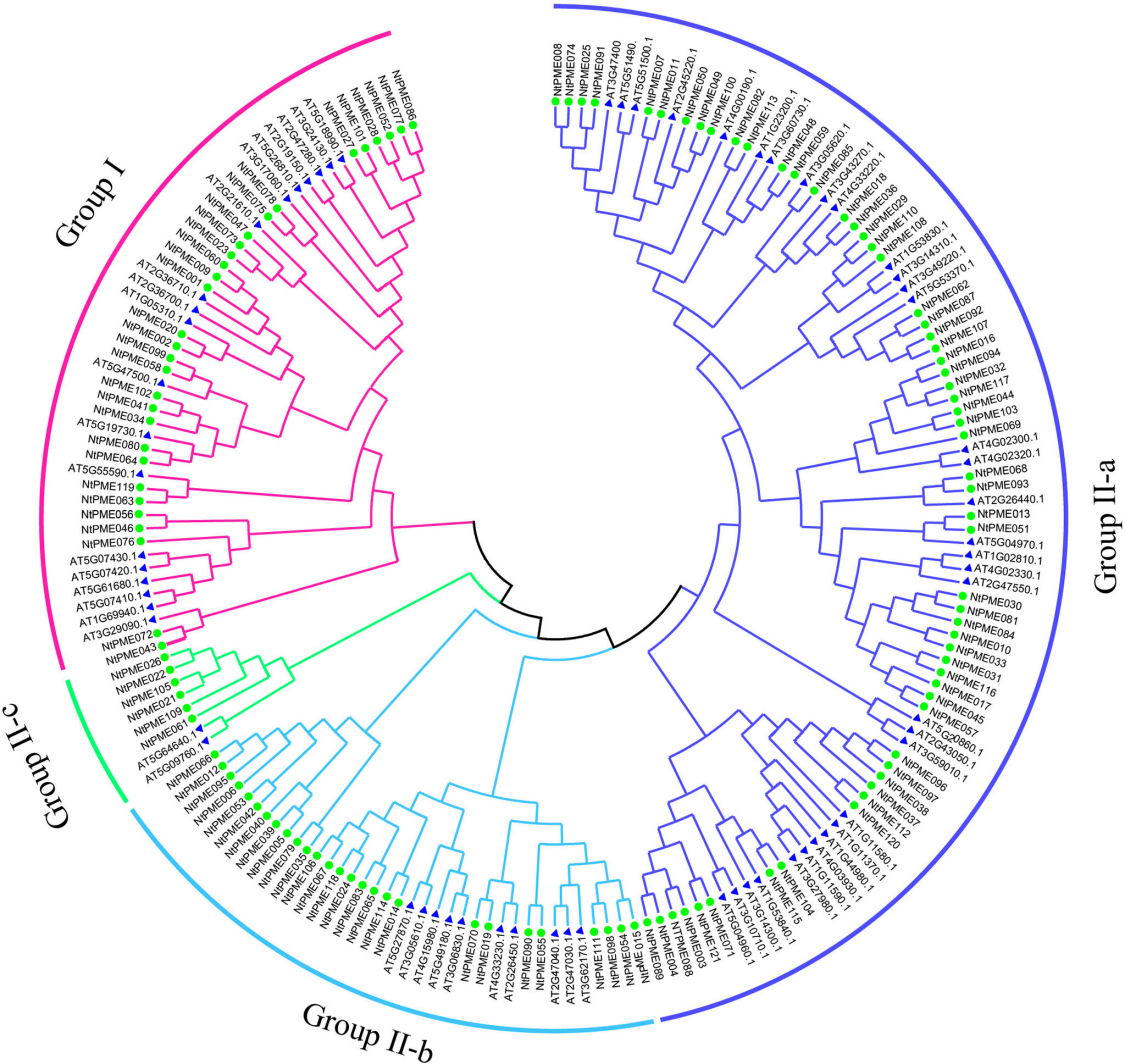
Phylogenetic relationship dendrogram of NtPME members in tobacco. The phylogenetic tree was generated by NtPME proteins of tobacco and *Arabidopsis* using the Neighbor-Joining method.

To better elucidate the evolutionary relationship of PME family members in tobacco, the MEGA7.0 software was employed to construct a neighbor-joining (NJ) tree consisting of tobacco (121 members) and *Arabidopsis* (66 members). The 121 NtPME proteins could be clustered into two groups(Group I and Group II) according to the PME or PMEI domain contained in these proteins with previous support (Li et al., 2020). Group II was further subdivided into three subclasses Group II-a, Group II-b, and Group II-c in this neighbor-joining (NJ) tree (**Figure 1**). Group I included 30 NtPME members with only PME domains, accounting for 24.8% of the total numbers. Seven of the members contained two PME domains, including NtPME20, NtPME23, NtPME27, NtPME60, NtPME63, NtPME99. However, the remaining others contained only one PME domain. Group II included 91 NtPME members with simultaneously PME and PMEI domains, accounting for 75.2% of the total numbers. To further understand the number and proportion of *NtPME* gene family members in plant genomes, six representative plant species were counted the members and the proportion of PME and PMEI, such as tobacco, *Arabidopsis*, rice, potato, Asian cotton, and *physcomitrella patens* (**Table 1**). As shown in **Table 1**, the number of PME proteins in Group I was generally less than that in Group II in plant species except for rice. The number of genes in Group I is approximately the same in different plant species. Notably, the 13 PME numbers in *physcomitrella patens* all belong to Group I. This result indicated that the PME genes of Group II may appear after the evolutionary differentiation of bryophytes plants. However, the number of PME proteins of Group II in tobacco far exceeded other species, which may be since tobacco is allopolytraploid.

**Table 1:** Statistics on the number of *PME* genes in six plant species.

Plant species	Group I (PME domain)		Group II (PME and PMEI domain)		Total number
	Number of genes	The proportion of total number (%)	Number of genes	The proportion of total number (%)	
*Arabidopsis*	23	34.8	43	65.2	66
Rice	23	56.1	18	43.9	41
Potato	26	48.1	28	51.9	54
Asian cotton	33	41.3	47	58.7	80
*physcomitrella patens*	13	100.0	0	0	13
Tobacco	30	24.8	91	75.2	121

### Chromosomal distribution and duplication events

**Figure 2:**
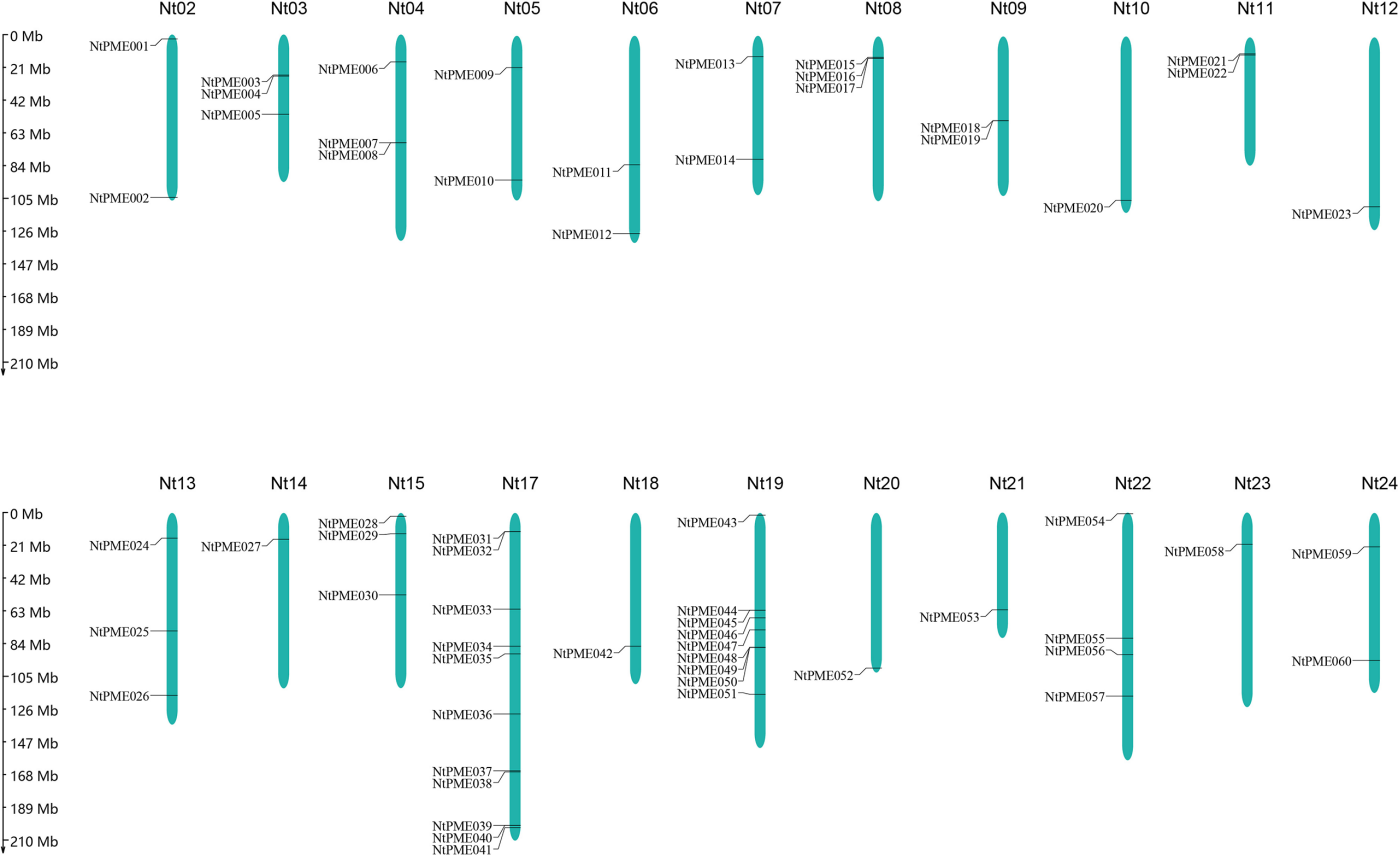
Distribution of 121 *NtPME* genes on the 24 chromosomes in tobacco.

In this study, a total of 121 *NtPMEs* were identified in tobacco and the information on chromosomal distribution was shown in **Figure 2**. The results showed that only 60 *NtPMEs* were mapped on 22 chromosomes, and the remaining genes were located on scaffolds. Chromosome 17 contained the largest number of *NtPMEs* (11 *NtPMEs*), while the other remaining chromosomes harbored less than four *NtPMEs*, for instance, chromosome 2 and chromosome 12. In this study, tandem events were carefully screened in the tobacco genome and indicated that no many tandem events were found between these *NtPME* genes.

**Figure 3:**
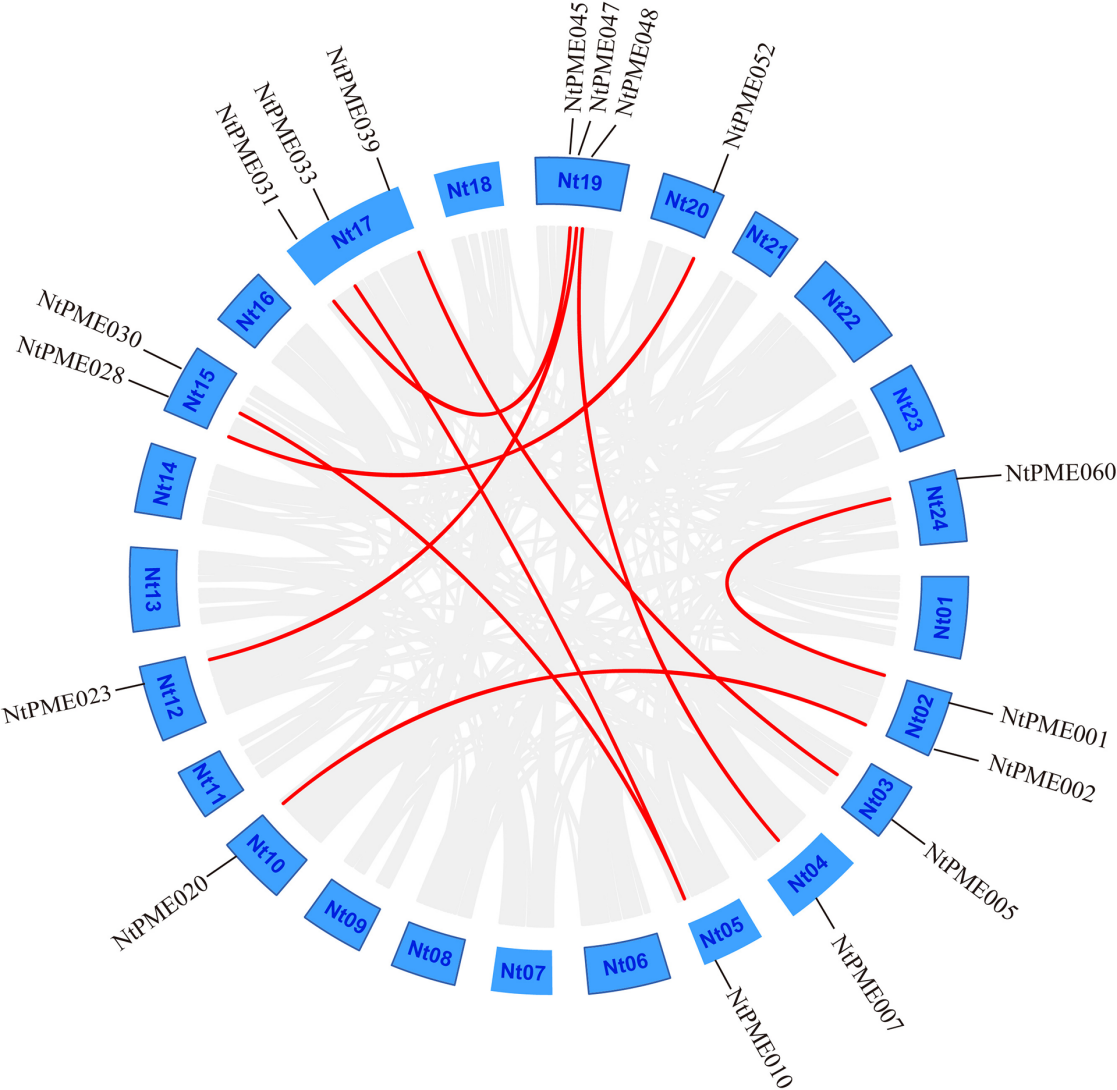
Segmental duplication analysis of the *NtPME* genes. The putative segmental duplication pairs are displayed by the gray line, whereas the *NtPME* segmental duplication pairs are revealed by the red line.

In addition, the segmental duplication analysis of *NtPMEs* was visualized using the MCScanX program (Cao et al., 2016). Notably, nine segmental duplication pairs in 17 *NtPMEs* were identified (**Figure 3**) and the details of these genes were listed in **Supplementary Table S3**. Four pairs of segmental duplication occurred in Group I (*NtPME001*/*NtPME060*, *NtPME002*/*NtPME020*, *NtPME023*/*NtPME047* and *NtPME028*/*NtPME052*) and Group II-a (*NtPME007*/*NtPME048*, *NtPME10*/*NtPME30*, *NtPME10*/*NtPME33* and *NtPME031*/*NtPME045*), respectively. However, only one pair of duplications (*NtPME005*/*NtPME039*) occurred in Group II-b. These results suggested that about 14% of the *NtPMEs* may be generated by segmental duplication events, which played a major role in the expansion of the *NtPMEs* family in tobacco. In addition, the ratio between non-synonymous and synonymous substitutions (Ka/Ks) can be used to estimate the purifying selection, neutral mutations, and beneficial mutations. In the present study, the Ka/Ks ratios of 9 segmental duplication gene pairs were calculated and the results showed that all the Ka/Ks ratios were less than 1, revealing that these *NtPMEs* may have undergone purifying selective pressure in the process of evolution.

### Syntenic analysis of *NtPME* genes

**Figure 4:**
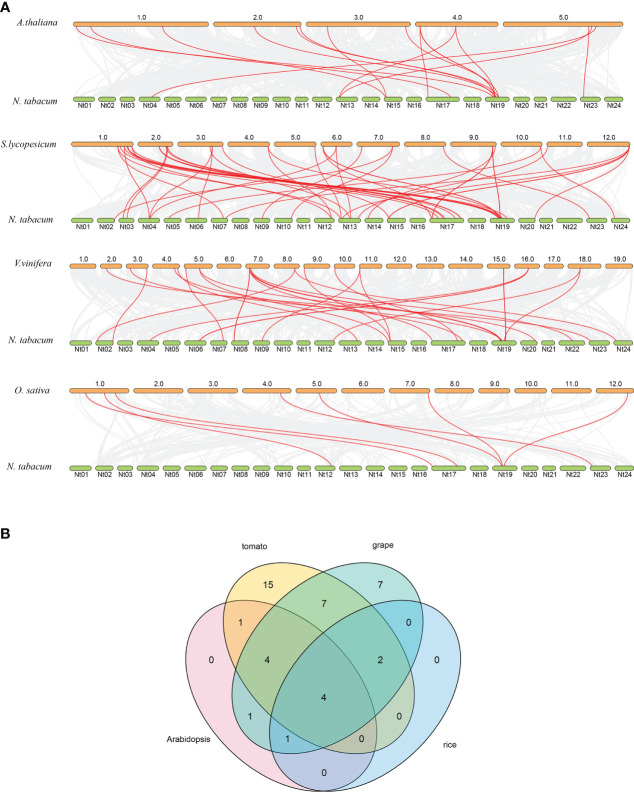
Syntenic relationship analysis dendrogram of *PME* genes among tobacco and four other plant species. **(A)** The collinear pairs between tobacco and four other plant species are displayed by the gray line, whereas the collinear *PME* gene pairs are revealed by the red color; **(B)** The *PME* genes formed the syntenic pairs between tobacco and all the other four plant species.

To further understand the genetic relationship of *NtPMEs*, the synthetic analysis of *PMEs* from dicotyledonous plants (*Arabidopsis*, tomato, and grape) and monocotyledonous plants (rice) were performed with *NtPMEs* (**Figure 4A**). The results showed that the numbers of predicted collinear pairs between tobacco and tomato were 46, followed by grape (28), *Arabidopsis* (16), and rice (7), respectively. The collinearity of tobacco with the other four plants indicated that a close genetic relationship existed between *NtPMEs* and tomato *PME* genes. Meanwhile, only four *NtPMEs* (*NtPME32*, *NtPME44*, *NtPME45*, *NtPME58*) formed collinear pairs with *PME* genes from all the other four plants, suggesting that these *NtPMEs* may have existed before the divergence of these plant species. (**Figure 4B** and **Supplementary Table S4**)

### Gene structure, multiple sequence alignment, and conserved motif analysis

**Figure 5:**
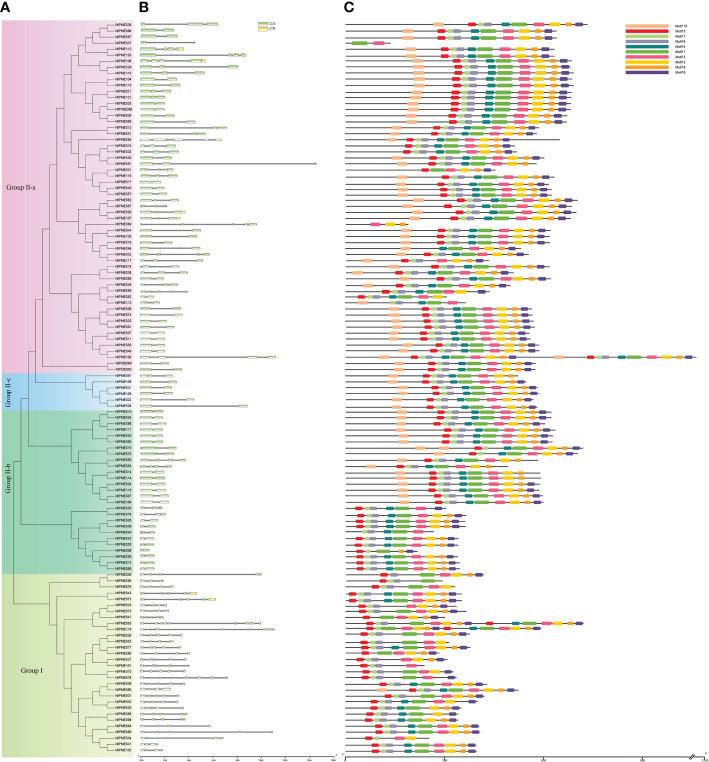
Gene structure and motif organizations analysis of NtPME members in tobacco. **(A)** The phylogenetic analysis tree of *NtPME* genes; **(B)** Gene structure of *NtPME* genes. Introns and exons are revealed by grey lines and light green boxes, respectively; **(C)** Distribution of conserved motifs in NtPME proteins. Different color boxes highlight different motifs.

To further analyze the structural diversity of *NtPMEs* in tobacco, the neighbor-joining phylogenetic tree had been constructed with only NtPME members (**Figure 5A**). Each NtPME was clustered into different groups, consistent with its distribution in the evolutionary tree of *Arabidopsis* and tobacco, suggesting that the construction of the neighbor-joining phylogenetic tree is reasonable. In addition, the number and arrangement of exon-intron in *NtPMEs* were also identified to provide insights into the evolution of these genes in tobacco (**Figure 5B**). The results showed that these *NtPMEs* clustered into the same group generally have almost identical exon-intron structures. The coding sequences of the *NtPMEs* were interrupted by introns, and the number of exons varied from one to 11. Notably, *NtPMEs* in Group I generally have more introns than that *NtPMEs* in Group II. In Group I, only one *NtPME* gene (*NtPME060*) has two introns, and the remaining *NtPMEs* contained more than three introns, of which *PME063* contained 10 introns. In Group II, there are 50 *NtPMEs* with only one intron (*NtPME017*, *NtPME030*, *NtPME045*, *NtPME068*, *NtPME093*, etc) and most of the remaining genes contain 2-3 introns (*NtPME010*, *NtPME031*, *NtPME033*, *NtPME071*, etc). These results indicate that the structure of *NtPMEs* in Group II is relatively simple compared to Group I. The effect of different intron numbers in *NtPMEs* on the function of these genes is a question worthy of further research.

The PME proteins generally have five conserved domains, including Region I (GxYxE), Region II (QAVAxR), Region III (QDTL), Region IV (DFIFG), and Region V (YLGRxWx). To further investigate the conserved domains of NtPME proteins in tobacco, 121 NtPME proteins were subjected to multiple sequence alignments of amino acid sequences, and the basic region was counted in **Supplementary Table S5**. As the results show that not all NtPME proteins contained all the conserved structural regions. There were 28 NtPME proteins contained less than five conserved structural regions, such as the NtPME006 protein sequence only contained four conserved structural regions. Moreover, there are ten conserved motifs (motif 1-motif 10) were analyzed by using MEME. The NtPME numbers in the same branch of the evolutionary tree usually have similar motif types and orders (**Figure 5C**). In addition, the motif 7, motif 4, and motif 2 correspond to Region I (GxYxE), Region II (QAVAxR), and V (YLGRxWx) conserved domains, respectively. Motif 1 contains Region III (QDTL) and Region IV (DFIFG) conserved domains.

### Promoter analysis of *NtPME* genes

In general, it was found that various *cis*-elements were identified in the tobacco *NtPMEs* promoters (**Supplementary Table S6**). Furthermore, 11 *cis*-elements involved in different hormone responses, developmental processes, and stress responses were selected for further analysis (**Figure S1**). The result showed that there were many *cis*-acting elements related to stress response in promoter regions of *NtPME* genes, for instance, ARE (anaerobic induction element), LTR (low-temperature-responsive element), MBS (MYB binding site), TC-rich repeats (stress-responsive element) and WUN-motif (wound-responsive element). The genes containing these *cis*-acting elements accounted for 88%, 49%, 50%, 45%, and 52% of all *NtPMEs*, respectively. In addition, the hormone-response elements were also identified in promoter regions, including ABRE, AuxRR-core, TCA-element, and CGTCA-motif. These *cis*-elements are potential responses to abscisic acid, auxin, ethylene, and methyl jasmonate, respectively.

### Gene expression patterns of *NtPME* genes

To preliminarily investigate the significant roles of *NtPMEs* in tobacco growth and development, the transcript data of *NtPMEs* in three tissues (root, shoot, and shoot apex) were downloaded from the GEO database and analyzed (**Figure S2**). The results showed that 2/3 of 121 *NtPME* genes were detected to be expressed in at least one tested tissue, and seven *NtPMEs* (*NtPME029*, *NtPME032*, *NtPME038*, *NtPME092*, *NtPME096*, *NtPME108* and *NtPME116*) were highly expressed in all three tested tissues. Furthermore, many *NtPMEs* were exclusively expressed in a tissue-specific manner. For instance, *NtPME038*, *NtPME073*, and *NtPME096* genes were the most exclusively expressed in the roots. *NtPME046* and *NtPME103* were detected highly expressed in the shoot apex. In addition, high-level expressions of *NtPME062*, and *NtPME093* were detected in the shoot and shoot apex.

To verify the reliability of *NtPMEs* RNA sequencing data analysis, we selected representative *NtPME* genes in different groups to carry out qRT-PCR analysis (**Figure S3**). *NtPME058*, *NtPME062*, and *NtPME093* were detected highly expressed in the shoot apex and shoot, respectively, which were consistent with the RNA sequencing data. In Group I, *NtPME072* was observed to be highly expressed in both leaves and flowers, while *NtPME47* was highly expressed in both roots and flowers. In Group II-a, *NtPME108* was detected highly expressed in the shoot, shoot apex, and flowers, whereas *NtPME029* was found to be highly expressed in root and shoot apex. Furthermore, *NtPME024* and *NtPME106*, classified into Group II-b, were found to exhibit similar expression patterns to *NtPME082* (Group II-a) and *NtPME056* (Group II-c), which were highly expressed in flowers. The results showed slight differences between qRT-PCR results and RNA sequencing data analysis, which might be due to the harvest of different sample methods and tissue development status.

**Figure 6:**
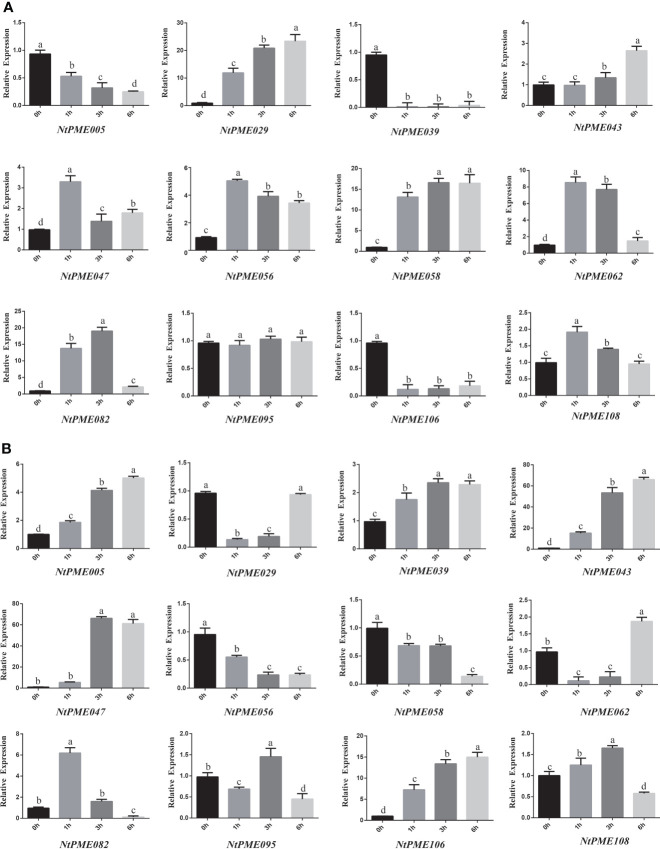
The expression patterns of *NtPME* genes under **(A)** Abscisic Acid (ABA) and **(B)** NaCl. For abiotic stress (salt and ABA) treatments, the seedlings were treated with 50 µM Abscisic Acid (ABA) or 150 mM NaCl, and then harvested at 0, 1, 3, or 6 h after treatment. Three biological replicates were performed. Values with superscript letters a, b, c, and d are significantly different across columns (*P* < 0.005).

In addition, to further investigate the expression patterns of *NtPMEs* under abiotic stress, the expression levels of 12 *NtPME* genes were determined under ABA (**Figure 6A**) and salt (**Figure 6B**) treatments. In Group I, *NtPME043*, *NtPME047*, *NtPME056*, and *NtPME058* were induced and up-regulated by ABA treatments, whereas these four genes were detected to exhibit different expression patterns under salt stress, *NtPME043* and *NtPME047* were induced and up-regulated, and *NtPME056* and *NtPME058* were down-regulated. Interestingly, the expression patterns of *NtPMEs* in Group II-a were different under ABA and salt treatments. It was found that ABA-induced and continually up-regulated *NtPME029* with the prolongation of treatment time, while the expression levels of *NtPME062*, *NtPME082*, and *NtPME108* first increased and then decreased with the increase of treatment time. Furthermore, with increasing treatment time, *NtPME029* and *NtPME062* were down-regulated by salt, while *NtPME082* and *NtPME108* behaved oppositely. In Group II-b, *NtPME005*, *NtPME039*, and *NtPME106* were found to induce by NaCl stress and down-regulated under ABA treatments. The expression of selected *NtPME* genes in NaCl and ABA treatment seemed to have opposite trend, suggesting that these genes may be involved in ABA pathway to response to NaCl abiotic stresses. How NtPME members including NtPME005 and NtPME039 involved in ABA and NaCl pathway will be the next questions to be addressed in this field.

### Subcellular localization analysis

**Figure 7:**
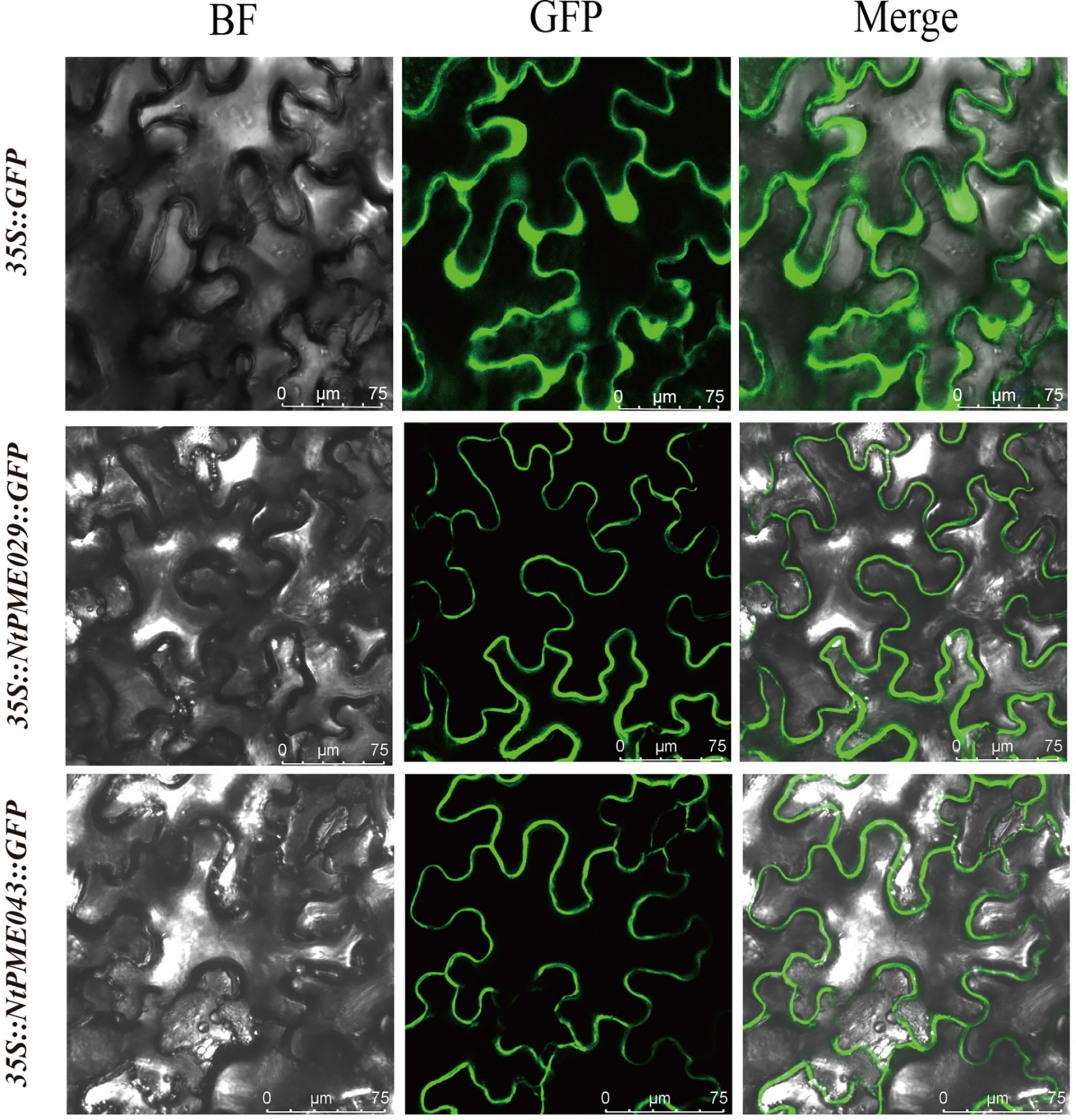
Subcellular localization of NtPME029 and NtPME043 proteins. The *NtPME029-GFP*, *NtPME043-GFP* fusion construct, and *GFP* gene all driven by the CaMV-35S promoter were transiently expressed in *N.benthamiana* leaves individually. BF, the signal of bright filed; GFP, the signal of GFP protein; Merge, the merge signal of GFP and BF signals. Scale bar = 75 µm.

To further investigate the potential functions of the *NtPMEs*, the NtPME029 and *NtPME043* were selected for subcellular localization analysis (**Figure 7**). The *p35S::NtPME029-GFP* and *p35S::NtPME043-GFP* constructs were introduced into the leaves of *Nicotiana benthamiana* and the *p35S:: GFP* as a contrast. The fluorescence signals showed that NtPME029 and NtPME043 may be the cell wall localization proteins.

### *NtPME* genes involvement in root development and salt tolerance of tobacco

In *Arabidopsis*, *AtPME3*-overexpressing plants showed longer root development compared to wild-type. In addition, *AtPME31* could be significantly induced under salt stress and enhance the salt stress tolerance of plants. *NtPME029* and *NtPME043*, were identified as homologues with *AtPME3* and *AtPME31*, respectively. Furthermore, *NtPME029* was highly expressed in roots, while *NtPME043* could be induced by salt treatments.

**Figure 8:**
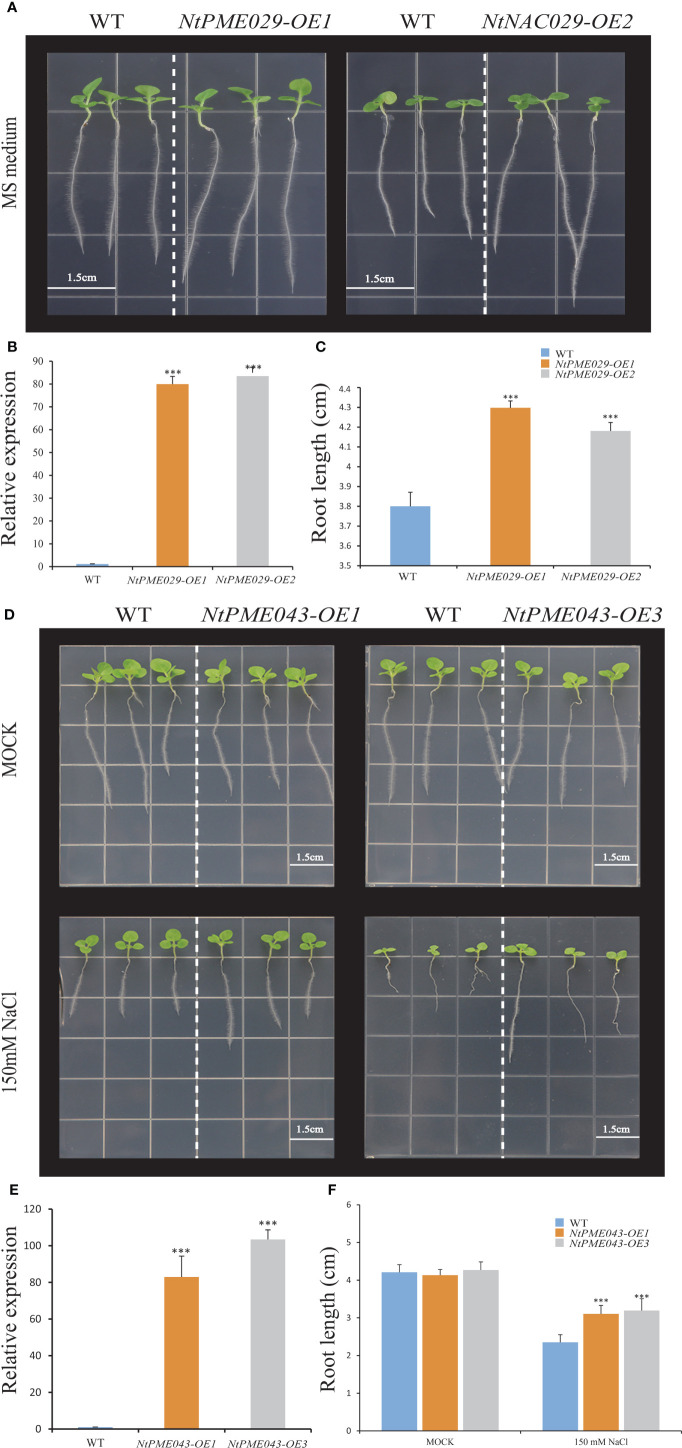
The over-expressing *NtPME029* and over-expressing *NtPME043* transgenic plants promote root growth and enhance salt stress, respectively. **(A, D)** Root growth of WT (wild type) and over-expressing *NtPME029* and over-expressing *NtPME043* lines under MS medium or MS contained 150 mM NaCl; **(B, E)** The expression level analysis of over-expressing *NtPME029* and over-expressing *NtPME043* tobacco plants, respectively; **(C, F)** Quantification and analysis of primary root length on media were retrieved from 27 plants of each genotype with three biological replicates. *** means *P* < 0.01 (Student’s *t*-test).

To further explore the function of the *NtPME029* gene in root development, the root elongation assay of wild-type (WT) and the *NtPME029*-overexpressed transgenic tobacco plants were assessed (**Figures 8A–C**). As result, the over-expressing *NtPME029* displayed longer root lengths than WT after three weeks of growth under normal conditions, suggesting that the *NtPME029* gene positively regulates the root development in tobacco.

To further analyze the function of the *NtPME043* gene in salt tolerance of tobacco, the root elongation assay of WT and the over-expressing *NtPME043* transgenic tobacco plants were also assessed (**Figures 8D–F**). The *NtPME043*-overexpressed plants displayed no significant differences in root length grown normal nutrient medium. However, the transgenic tobacco plants produced longer root lengths than WT after three weeks of growing under 150 mM NaCl plates. It indicated that the *NtPME043* gene could enhance salt tolerance when overexpressed in tobacco.

## Discussion

Previous studies reported that *PMEs* play critical roles in response to diverse abiotic stresses and plant development processes (Jeong et al., 2015; Duan et al., 2016; Zhang et al., 2019). In the present study, a total of 121 members of the *NtPME* genes were identified in the tobacco genome using BLASTP searches. Furthermore, the *NtPMEs* were analyzed using phylogeny, gene structure, motif organization, chromosomal distributions, duplication events, *cis*-elements, expression profiles, and potential functions.

The 121 NtPME members were divided into two groups according to the phylogeny analysis of *Arabidopsis* NtPME members. (Louvet et al., 2006). The NtPME members in the same group contained similar gene structure and motif organization consistent with the previous reports (Li et al., 2020), suggesting the evolutionary relationship and classification analysis of *NtPME* genes were reliable. Additionally, some *NtPME* genes of the same group contained similar *cis*-elements types, implying that they may reflect similar functions in plant development and abiotic stress responses. Interestingly, statistics on the number of *PME* genes in six plant species found the *PME* genes in tobacco significantly larger than in other plants (**Table 1**), probably due to tobacco being an allopolytraploid. Meanwhile, five higher plants all have Group I and Group II PME genes, while hyscomitrella patens only have Group I *PME* genes, implying that the *PME* genes of Group II appeared after the differentiation of moss and vascular plants, which is consistent with the study of Pelloux and Markovic et al., (Markovic and Janecek, 2004; Pelloux et al., 2007). The synthetic analysis of *NtPMEs* found four genes may have existed before the divergence of dicotyledonous and monocotyledonous plant species (**Figure 4**), suggesting that these genes were relatively conserved during evolution.

Previous studies suggested that the whole genome duplication events contribute to the expansion of gene families (Duan et al., 2016; Khan et al., 2019). In this study, the expansion of the tobacco *NtPME* gene family may be mainly attributable to segmental duplications, not tandem duplication. A total of nine segmental duplication pairs were identified in *NtPMEs*, with the Ka/Ks ratios of these duplication pairs being less than 1 (**Figure 3** and **Supplementary Table S3**), suggesting that these *NtPME*s might have undergone purifying selective pressure. Especially, *NtPME005* and *NtPME039* as duplicated gene pair was found to exhibit similar gene structure, conserved motif organization, and expression patterns (**Figures 5**, **6**), which indicates the similarity of their functions.

According to previous reports, several PME members affect the plant development in *Arabidopsis*. Specifically, *AtPME5* (*AT5G47500*) facilitates shoot development (Peaucelle et al., 2011), while *NtPME058* (Group II-a) as its syntenic pair gene in tobacco was detected to be highly expressed in the shoots and shoot apex, indicating that *NtPME058* may regulate the shoot development (**Figure S3**). In plants, floral development could affect seed formation, with implications for seed embryo development. In Group II-b, *AtPME58* reportedly played a significant role in seed maturation (Turbant et al., 2016). As tobacco homologs, *NtPME024* and *NtPME106*, are highly expressed in flowers. In Group I, *AtPME48* (*AT5G07410*) is highly expressed in the flower and functions to influence pollen development (Leroux et al., 2015). *NtPME043*, *NtPME056*, and *NtPME072* classified in the same group as *AtPME48* were both highly expressed in flowers, implying that these genes may also modulate floral development. The evidence indicates that *AtPME3* and *AtPME17* specifically regulate the root growth and development of *Arabidopsis* (Hewezi et al., 2008; Sénéchal et al., 2014). Notably, the *NtPME029* was highly expressed in the root and identified as homologues with *AtPME3*, suggesting that they might have similar biological functions in the root growth and development. The experiment of *NtPME029*-overexpressing also validated this idea (**Figures 6**, **8A, C**). Additionally, the promoter regions analysis of *NtPME029* was found to harbor ABRE and AuxRR-core elements, implying that this gene might participate in ABA and auxin pathways to mediate the root development. Whereas, in the same group, *NtPME082* was highly expressed in the flowers, indicating possible functional divergence between these *NtPME* genes.

Previous studies have indicated that *AtPME* genes mediate responses to various abiotic stresses. In Group II-b, *AtPME28* (*AT5G27870*) is induced by NaCl treatment to regulate salt stress (Qu et al., 2011). Consistent with *AtPME28*, the expression of *NtPME005*, *NtPME039* and *NtPME106* could also be induced under salt treatments, suggesting that these genes clustered in the same group may display similar functions in response to abiotic stress. *NtPME047* embedded low-temperature-responsive element (LTR) was closely related to *AtPME41* (*AT4G02330*), enhancing freezing tolerance (Qu et al., 2011), indicating that *NtPME047* may have a similar function under chilling stress. In-plant tissues, *PME* genes with the ABA response element (ABRE) can be induced by ABA treatment. In Group II-a, *AtPME34* (*AT3G49220*) is verified to respond to heat and salt stress through the signaling of the ABA (abscisic acid) pathway (Huang et al., 2017). *NtPM0E62* and *NtPME092*, containing ABRE elements, were detected to induce by both ABA and NaCl treatment, implying that they may be involved in the ABA synthesis pathway in response to salt stress. In Group I, *AtPME31* (*AT3G29090*) could be significantly induced under salt stress and positively modulate the expression levels of salt stress-induced genes to enhance the salt stress tolerance of plants (Yan et al., 2018). Its tobacco homolog, *NtPME043*, was also induced under salt stress and contained the stress-responsive element (TC-rich repeats) and MYB binding site (MBS) associated with stress response, implying that *NtPME043* may contribute to the salt stress response of tobacco. Furthermore, *NtPME043*-overexpressing analyses demonstrated that the *NtPME043* gene could enhance the salt tolerance of tobacco (**Figures 8D, F**). Additionally, the *NtPME029-GFP* and *NtPME043-GFP* fusion proteins located on the cell wall are consistent with previous evidence of PME proteins’ location (Yapo et al., 2007; Wolf et al., 2009).

## Conclusions

In this study, 121 *NtPME* genes were identified in the tobacco genome. The comprehensive analysis indicated that the *NtPME* gene family may play multiple roles in various biological processes of tobacco. The PME homologous members between *Arabidopsis* and tobacco displayed the conserved function in plant development and stress responses. Notably, the *NtPME029* was highly expressed in the root and *NtPME029*-overexpressing transgenic plants could promote root development. Furthermore, *NtPME043* could be induced by salt and ABA treatments, and the *NtPME043*-overexpressing transgenic plants significantly enhanced the salt-stress tolerance. This study could provide insight into the further biological functional characterization of *NtPME* genes in tobacco.

## Data availability statement

The original contributions presented in the study are included in the article/**Supplementary Material**. Further inquiries can be directed to the corresponding authors.

## Author contributions

JS, ZT, and XL conducted the research and participated in drafting the manuscript. The other authors assisted in data collection and analysis. MX and RX conceived this research, designed the experiments and drafted the manuscript. All authors contributed to the article and approved the submitted version.

## Funding

This work was supported by the Optimization of Quality Assurance Technology for China Tobacco special aroma 301 Variety and Its Application in Middle and High-end Cigarettes (Y02J202102); the Agricultural Science and Technology Innovation Program (ASTIP-TRIC02); the China Tobacco Genome Project [110202001029(JY-12)] and the China Tobacco Hunan Industrial Co., Ltd. Technology Project (KY2022YC0010).

## Conflict of interest

JS, ZT, SL, JW, ZH, HC, and RX were employed by China Tobacco Jiangsu Industrial Co., Ltd.

The remaining authors declare that the research was conducted in the absence of any commercial or financial relationships that could be construed as a potential conflict of interest.

## Publisher’s note

All claims expressed in this article are solely those of the authors and do not necessarily represent those of their affiliated organizations, or those of the publisher, the editors and the reviewers. Any product that may be evaluated in this article, or claim that may be made by its manufacturer, is not guaranteed or endorsed by the publisher.
