# Supplementary material for: Systematic analysis of the pectin methylesterase gene family in Nicotiana tabacum and reveal their multiple roles in plant development and abiotic stresses
Source: Front Plant Sci. 2022 Sep 28;13:998841. doi: 10.3389/fpls.2022.998841 (PMC9554592; doi:10.3389/fpls.2022.998841)
Supplement: Supplementary file 1 [file DataSheet_1.zip › Supplementary Table S1. The information of qRT-PCR primers used in this study.docx]

Supplementary Table S1. The information of qRT-PCR primers used in this study

| Gene | Forward primer | Reverse primer |
| --- | --- | --- |
| PME001 | GAGATTATTATTGCTCGTCGCC | GGCCTTTTGTACACTGCTAAAA |
| PME014 | ACTCGGGAGCTAAGTATCAATG | TTGCAGAAGCTTACGGTTACTA |
| PME022 | TACGCAGATAATAGGATCGAGC | CGAGTTCAAAAACGAGATGGTT |
| PME024 | TGCCAATGCTAAGAACATCAAC | CACGTTTTTAAGTCATCGGTGT |
| PME029 | CTCCAATCGTCAATTCTTCGTC | GTGTTTTGGTTGGGATCCTTAC |
| PME043 | TTTGAGCTTCACCATTGGAAAG | GTGTAAACATTTGGTGCGGATA |
| PME045 | GGAGTGTTAGAAATGGCCTAGT | CACCCTTTTGTGAACAAAGCTA |
| PME049 | AAGATCAAAAGCACAGTTGGTC | CATGTTTCGAGGTTGGTTAAGG |
| PME056 | GCCTTTGAAGACATTCCAAAGT | CATGTGCTGCTGTATTCTCAAA |
| PME058 | CATGTGCTGCTGTATTCTCAAA | ATTCTGGAGTATTGGCCCATAG |
| PME062 | CACATATATGGCACGGTAGACT | GAAACTCCCTTTAACAGCACTG |
| PME067 | TTACAAATTTGCATGTCCCTGG | AACTTGAAGAAGCCTACGAGAA |
| PME082 | GTCTTCACCACTGGCAGATATA | ATTCATGGTATTCTCGACCTCC |
| PME092 | TGCCACTCTTCTTTTAATTGCC | TTGAGACAGAGAGATGGGTAGA |
| PME106 | GGAGTTGGTGCAATCGTATTAC | GTCCAAGGTACGCTTTAATTGG |
| PME108 | TTATCGTTGCAGTTTCAAAGGG | GCTGCGTCTCCAAATATGAAAT |
| L25 | GAAGATTGAGGACAACAACACC | TCTTCACGGCATCCTTAATCTT |
