# Supplementary figures and images for: Systematic analysis of the pectin methylesterase gene family in Nicotiana tabacum and reveal their multiple roles in plant development and abiotic stresses
Source: Front Plant Sci. 2022 Sep 28;13:998841. doi: 10.3389/fpls.2022.998841 (PMC9554592; doi:10.3389/fpls.2022.998841)

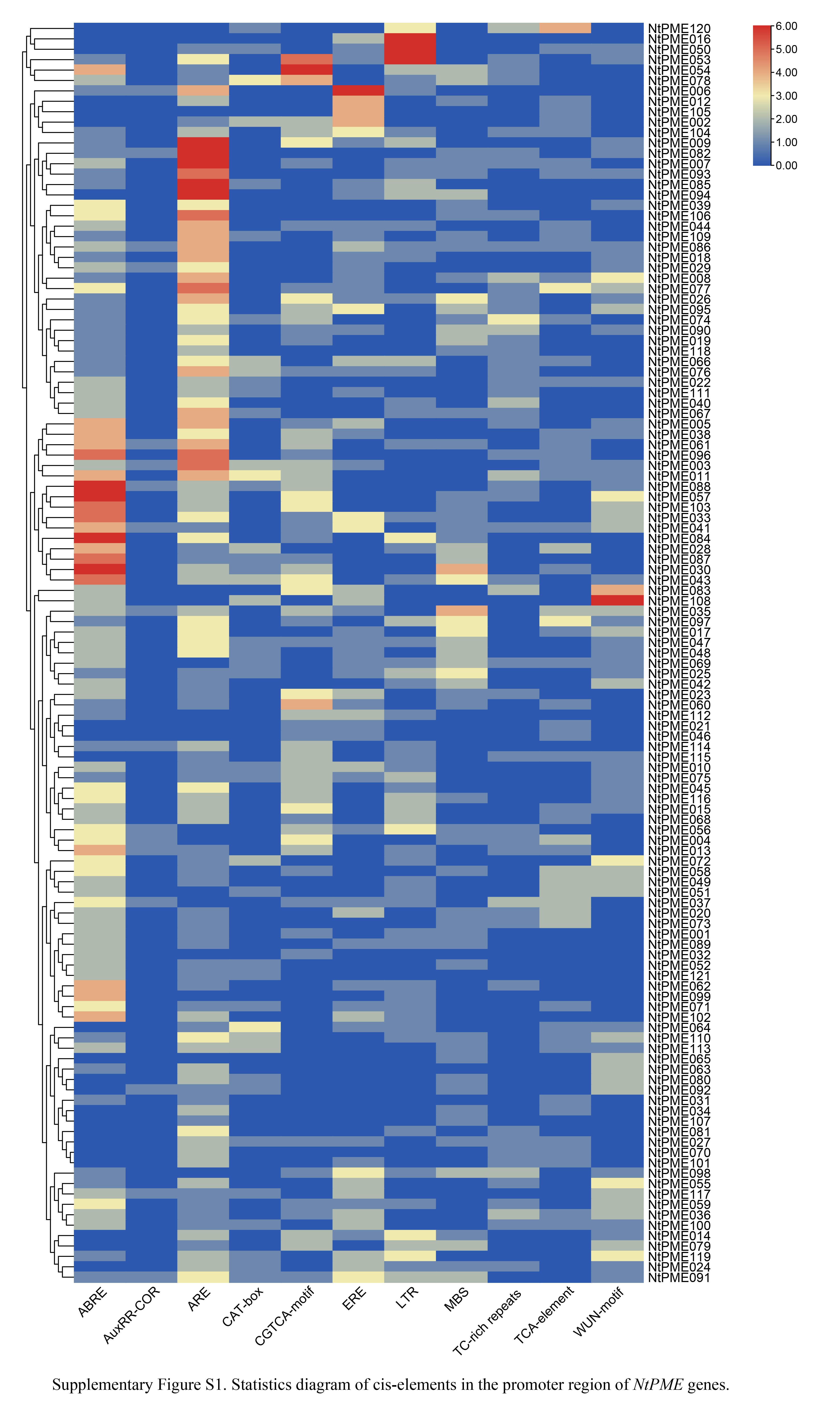

Supplement: Supplementary file 1 [file DataSheet_1.zip › Supplementary Figure S1. Statistics diagram of cis-elements in the promoter region of NtPME genes.jpg]

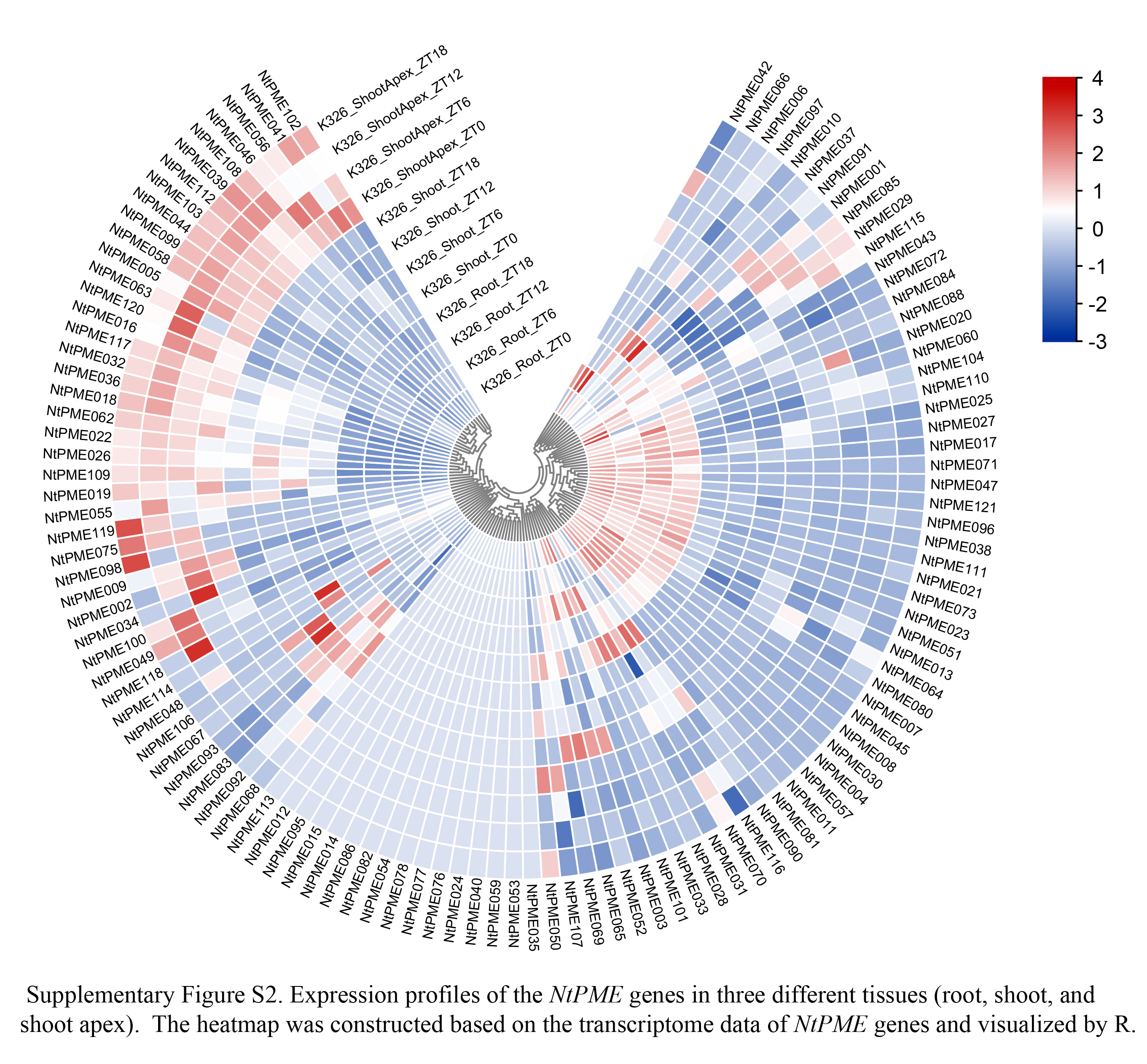

Supplement: Supplementary file 1 [file DataSheet_1.zip › Supplementary Figure S2. Expression profiles of the NtPME genes in three different tissues (root, shoot, and shoot apex)..jpg]

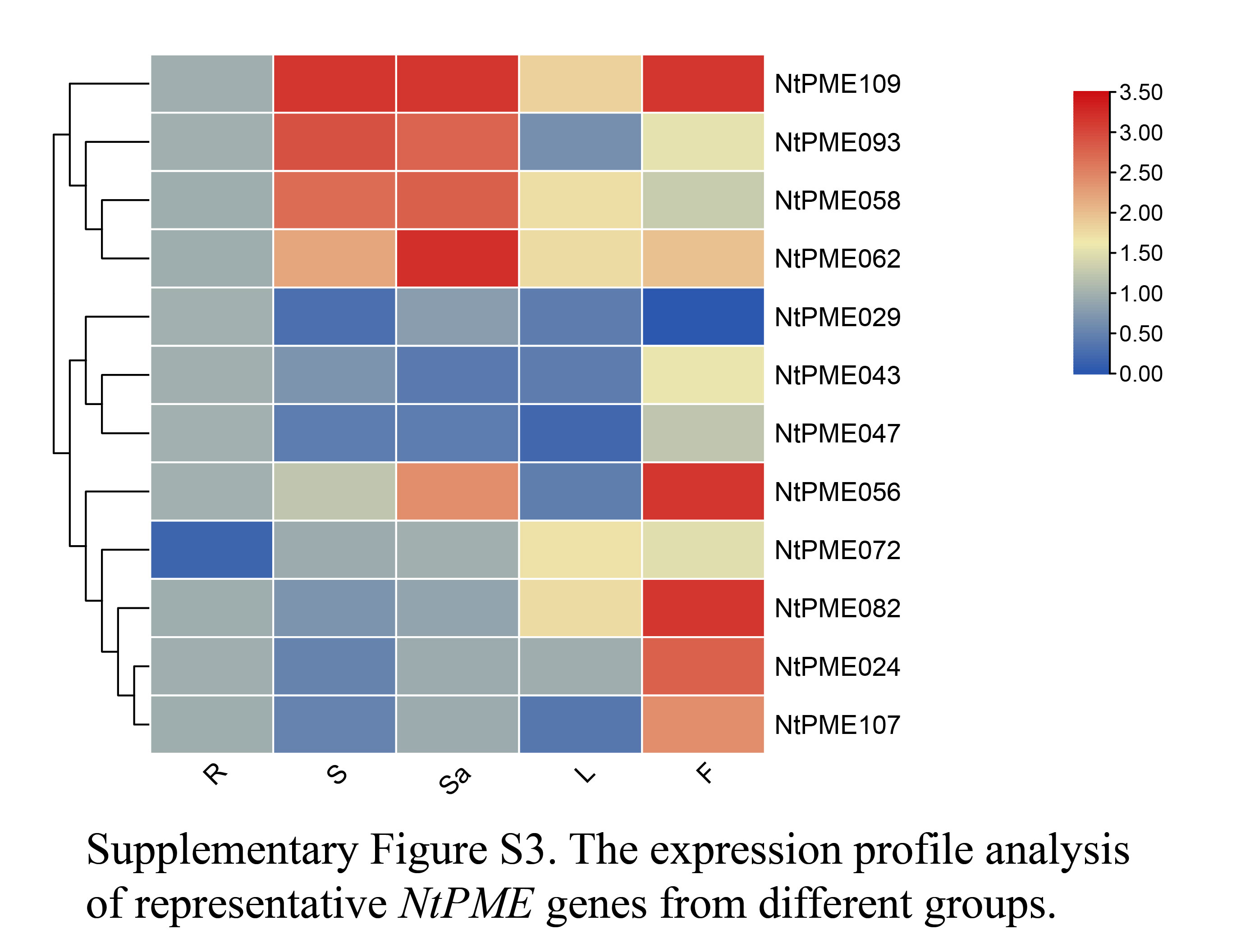

Supplement: Supplementary file 1 [file DataSheet_1.zip › Supplementary Figure S3. The expression profile analysis of representative NtPME genes from different groups-01.jpg]
