# Supplementary material for: Systematic analysis of the pectin methylesterase gene family in Nicotiana tabacum and reveal their multiple roles in plant development and abiotic stresses
Source: Front Plant Sci. 2022 Sep 28;13:998841. doi: 10.3389/fpls.2022.998841 (PMC9554592; doi:10.3389/fpls.2022.998841)
Supplement: Supplementary file 1 [file DataSheet_1.zip › Supplementary Table S3.The information of segmental duplication gene pairs in NtPME family genes.doc]

Supplementary Table S3. The information of segmental duplication gene pairs

in NtPME family genes

| Duplicated pairs | Ka | Ks | Ka/Ks | Purifying selection | Duplicate  type |
| --- | --- | --- | --- | --- | --- |
| NtPME001-NtPME060 | 0.119344660 | 0.460228547 | 0.259316074 | Yes | Segmental |
| NtPME002-NtPME020 | 0.040751464 | 0.178861276 | 0.227838384 | Yes | Segmental |
| NtPME005-NtPME039 | 0.036026937 | 0.101654763 | 0.354404810 | Yes | Segmental |
| NtPME007-NtPME048 | 0.447770194 | 1.695237027 | 0.264134270 | Yes | Segmental |
| NtPME010-NtPME030 | 0.255424520 | 2.801152174 | 0.091185521 | Yes | Segmental |
| NtPME010-NtPME033 | 0.029949978 | 0.073070183 | 0.409879608 | Yes | Segmental |
| NtPME023-NtPME047 | 0.428354236 | 2.191579698 | 0.195454556 | Yes | Segmental |
| NtPME028-NtPME052 | 0.026264270 | 0.060141496 | 0.436707960 | Yes | Segmental |
| NtPME031-NtPME045 | 0.238703604 | 0.938638019 | 0.254308476 | Yes | Segmental |
