# Supplementary material for: Systematic analysis of the pectin methylesterase gene family in Nicotiana tabacum and reveal their multiple roles in plant development and abiotic stresses
Source: Front Plant Sci. 2022 Sep 28;13:998841. doi: 10.3389/fpls.2022.998841 (PMC9554592; doi:10.3389/fpls.2022.998841)
Supplement: Supplementary file 1 [file DataSheet_1.zip › Supplementary Table S5. Amino acid sequence alignment results of.doc]

Supplementary Table S5. Amino acid sequence alignment results of

PME gene family in Tobacco

| Gene name | Region I  (GxYxE) | Region II  (QAVAxR) | Region III  (QDTL) | Region IV  (DFIFG) | Region V  (YLGRxWx) |
| --- | --- | --- | --- | --- | --- |
| NtPME001 | GTYKE | QAVAVR | QDTL | DFIFG | WLGRPWG |
| NtPME002 | GTYKE | QAVALR | QDTL | DFIFG | YLGRPWK |
| NtPME003 | GVYQE | QAVALM | QDTL | DFIFG | YLGRPWK |
| NtPME004 | GVYQE | QAVALR | QDTL | DFIFG | YLGRPWK |
| NtPME005 | GTYNE | QSAALT | QDTL | DFISG | FLGRPWK |
| NtPME006 | GMYGC | QAVASR | QDTL | DFICG | ------- |
| NtPME007 | GVYRE | QAVALR | QDTL | DFIFG | YLGRPWK |
| NtPME008 | GVYRE | QAVALR | QDTL | DFIFG | YLGRPWQ |
| NtPME009 | GTYRE | QAVALR | QDTL | DFIFG | WLGRPWG |
| NtPME010 | GVYEE | QAVALR | QDTL | DFIFG | YLGRPWK |
| NtPME011 | GAYRE | QAVALR | QDTL | DFIFG | YLGRPWK |
| NtPME012 | GTYYE | QAVALR | QDTL | DFICG | YLGRPWG |
| NtPME013 | GYYEE | QAVALR | QDTL | DFIFG | FLGRPWH |
| NtPME014 | GIYKE | QAVALR | QDTL | DFIFG | YLGRPWK |
| NtPME015 | GIYEE | QAVALR | QDTL | DFIFG | YLGRPWK |
| NtPME016 | GSYFE | QAVALR | QDTL | DFIFG | YLGRPWK |
| NtPME017 | GVYEE | QAVAVR | QDTL | DFIFG | YLGRPWK |
| NtPME018 | GIYNE | QAVAFR | QDTL | DFIFG | YLGRPWK |
| NtPME019 | GVYEE | QAVALR | QDVL | DIISG | YLGRPWK |
| NtPME020 | GIYFE | QAVALR | QDTL | ----- | YLGRPWK |
| NtPME021 | GLYEE | QAVAFR | QDTL | DFICG | YLGRPWK |
| NtPME022 | GLYDE | QAVAFR | QDTL | ----- | YLGRPWK |
| NtPME023 | GLYDE | KAVALR | QDTL | ----- | ILGRPWG |
| NtPME024 | GVYNE | QAVALR | QDTL | DFIFG | YLGRPWK |
| NtPME025 | GVYRE | QAVALR | QDTL | DFIFG | YLGRPWQ |
| NtPME026 | GLYDE | QAVALR | QDTL | DFIFG | YLGRPWK |
| NtPME027 | GLYKE | ------ | ---- | DFIFG | FLGRPWR |
| NtPME028 | GQYRE | ------ | QDTL | DFIFG | YLGRPWK |
| NtPME029 | GVYKE | QAVALR | QDTL | DFIFG | YLGRPWK |
| NtPME030 | GVYQE | QAVAVR | QDTL | DFIFG | YLGRPWK |
| NtPME031 | ----- | ------ | QDTL | DFIFG | YLGRPWK |
| NtPME032 | GTYFE | QAVALR | QDTL | ----- | YLGRPWK |
| NtPME033 | GVYEE | QAVALR | QDTL | DFIFG | YLGRPWK |
| NtPME034 | SIYRD | QAVALR | ---- | DFIFG | FLGRPWK |
| NtPME035 | GIYNE | QAVAVS | QDTL | DFIFG | ------- |
| NtPME036 | GIYNE | QAVALR | QDTL | DFIFG | YLGRPWK |
| NtPME037 | ----- | QAVALR | QDTL | DFIFG | ------- |
| NtPME038 | GIYKE | QAVALR | QDTL | DFIFG | YLGRPWK |
| NtPME039 | GPYNE | QSAALT | QDTL | DFISG | YLGRPWK |
| NtPME040 | ANYSD | QAAALT | QDTL | DFIFG | YLGRPWK |
| NtPME041 | GIYRE | QAVALR | QDTL | DFIFG | YLGRPWG |
| NtPME042 | GTYNE | QAVALR | QDTL | DFICG | YLGRPWG |
| NtPME043 | GIYKE | QAVALR | QDTL | DFIFG | HLGRPWG |
| NtPME044 | GAYFE | QAVAVR | QDTL | DFIFG | YLGRPWK |
| NtPME045 | GVYEE | QAVAVR | QDTL | DFIFG | YLGRPWK |
| NtPME046 | ----- | QAVALR | QDTL | DFIFG | YLGRPWK |
| NtPME047 | ----- | ------ | ---- | ----- | ALGRPWG |
| NtPME048 | GVYRE | QAVALR | QDTL | DFIFG | YLGRPWK |
| NtPME049 | GTYSE | QAVALR | QDTL | DFIFG | YLGRPWK |
| NtPME050 | GIYNE | QAVALR | QDTL | DFIFG | YLGRPWK |
| NtPME051 | GYYEE | QAVAFR | QDTL | DFIFG | FLGRPWH |
| NtPME052 | GQYRE | ------ | QDTL | DFIFG | FLGRPWR |
| NtPME053 | GTYHE | QAVALV | QDTL | DFIFG | YLGRPWK |
| NtPME054 | GIYEE | QAVALR | QDTL | DFIFG | YLGRPWK |
| NtPME055 | GIYDE | QAVALR | QDTL | DFIFG | YLGRPWK |
| NtPME056 | GVYRE | QAVALR | QDTL | DFIFG | ------- |
| NtPME057 | GVYEE | QAVAVR | QDTL | DFIFG | YLGRPWK |
| NtPME058 | GVYIE | QAVALR | QDSL | DFIFG | ------- |
| NtPME060 | GTYKE | QAVALR | QDTL | DFIFG | WLGRPWG |
| NtPME059 | GVYRE | QAVALR | QDTL | DFIFG | YLGRPWK |
| NtPME061 | GLYNE | QAVAFR | QDTL | DFIFG | YLGRPWK |
| NtPME062 | GRYEE | QAVALR | QDTL | DFIFG | YLGRPWK |
| NtPME063 | GAYDE | QAVALR | QDTL | DFICG | FLGRPWG |
| NtPME064 | GVYTE | QAVAFR | QDTL | DFIFG | YLGRPWG |
| NtPME065 | GIYNE | QAVAVR | QDTL | DFIFG | YLGRPWK |
| NtPME066 | GTYYE | QAVALR | QDNL | DFICG | FLGRPWG |
| NtPME067 | GEYKE | QAVALR | QDTL | ---- | YLGRPWK |
| NtPME068 | GVYQE | QAVALR | QDTL | DFIFG | FLGRPWR |
| NtPME069 | ----- | ------ | ---- | ----- | CLGRPWK |
| NtPME070 | GVYKE | QAVALR | QDTL | DFISG | YLGRPWK |
| NtPME071 | GIYKE | QAVALM | QDTL | DFIFG | FLGRPMK |
| NtPME072 | GVYKE | QAVALR | QDTL | DFIFG | HLGRPWG |
| NtPME073 | GIYRE | KAVALR | QDTL | DFICG | ILGRPKG |
| NtPME074 | GVYRE | QAVALR | QDTL | DFIFG | YLGRPWQ |
| NtPME075 | GTYRE | ------ | QDTL | DFIFG | YLGRPWK |
| NtPME076 | GNYTE | QAAALR | QDTL | DFIFG | YLGRPWK |
| NtPME077 | GKYRE | QAVALR | QDTL | DFIFG | FLGRPWR |
| NtPME078 | GTYRE | ------ | QDTL | DFIFG | YLGRPWK |
| NtPME079 | GFYNE | QAVAVS | QDTL | DFIFG | YLGRPWK |
| NtPME080 | GVYTE | QAVAVR | QDTL | DFIFG | YLGRPWG |
| NtPME081 | GVYQE | QAVALR | QDTL | DFIFG | YLGRPWK |
| NtPME082 | GNYTE | QAVALR | QDTL | DFIFG | YLGRPWK |
| NtPME083 | GIYHE | QAVALR | QYTL | DFIFG | YLGRPWK |
| NtPME084 | GIYHE | QAVALR | QDTL | DFIFG | YLGRPWK |
| NtPME085 | GIYHE | QAVALR | QDTL | DFIFG | YLGRPWK |
| NtPME086 | ----- | AAVAAM | QDTL | DFIFG | FLGRPWR |
| NtPME087 | GVYDE | QAVALR | ---- | ----- | ------- |
| NtPME088 | GVYVE | QAVALR | QDTL | DFIFG | YLGRPWK |
| NtPME089 | GVYVE | QAVALR | QDTL | DFIFG | YLGRPWK |
| NtPME090 | GIYDE | QAVALR | QDTL | DFIFG | YLGRPWK |
| NtPME091 | GVYRE | QAVALR | QDTL | DFIFG | YLGRPWQ |
| NtPME092 | GRYEE | QAVALR | QDTL | DFIFG | YLGRPWK |
| NtPME093 | GVYEE | QAVALR | QDTL | DFIFG | YLGRPWK |
| NtPME094 | ----- | QAVTLR | QDTL | DFIFG | YLGRPWK |
| NtPME095 | GTYYE | QAVALR | QDTL | DFICG | YLGRPWG |
| NtPME096 | GIYKE | QAVALR | QDTL | DFICG | YLGRPWK |
| NtPME097 | GIYKE | QAVALR | QDTL | DFIFG | YLGRPWK |
| NtPME098 | GIYNE | QAVALR | QDTL | DFIFG | FLGRPWK |
| NtPME099 | GYYIE | QAVAFR | QDTL | DFIFG | ------- |
| NtPME100 | GIYSE | QAVALR | QDTL | DFIFG | YLGRPWK |
| NtPME101 | GLYKE | ------ | ---- | DFIFG | FLGRPWR |
| NtPME102 | GIYRE | QAVALR | QDTL | DFIFG | YLGRPWK |
| NtPME103 | GAYYE | QAVAVR | QDTL | DFIFG | YLGRPWK |
| NtPME104 | GEYVE | QAVAMR | QDTL | DFIFG | YLGRPWK |
| NtPME105 | GLYEE | QAVAFR | QDTL | DFIFG | YLGRPWK |
| NtPME106 | GEYKE | QAVALR | QDTL | DFIFG | YLGRPWK |
| NtPME107 | GRYEE | QAVALR | QDTL | DFIFG | YLGRPMK |
| NtPME108 | GVYRE | QAVALR | QDTL | DFIFG | YLGRPWQ |
| NtPME109 | GLYKE | QAVAFR | QDTL | DFIFG | FLGRPWK |
| NtPME110 | GVYKE | QAVALR | QDTL | DFIFG | YLGRPWK |
| NtPME111 | GVYNE | QAVALR | QDTL | DFIFG | YLGRPWK |
| NtPME112 | GIYKE | QAVALR | QDTL | DFIFG | YLGRPWK |
| NtPME113 | GNYTE | QAVALR | QDTL | DFIFG | YLGRPWK |
| NtPME114 | GIYKE | QAVALR | QDTL | DFIFG | FLGRPWK |
| NtPME115 | GEYVE | QAVAMR | QDTL | DFIFG | YLGRPWK |
| NtPME116 | GVYEE | QAVAVR | ---- | DFIFG | YLGRPSK |
| NtPME117 | GTYRE | ------ | QDTL | ----- | ------- |
| NtPME118 | GVYNE | QAVALR | QDTL | DFIFG | YLGRPWK |
| NtPME119 | ----- | QAVAVR | QDTL | DFIFG | FLGRPWG |
| NtPME120 | GTYKE | QAVALR | QDTL | DFIFG | YLGRPWK |
| NtPME121 | GIYKE | QAVALM | QDTL | DFIFG | YLGRPWK |
